# Impaired health-related quality of life, and depressive symptoms in a cohort of healthy adults with symptoms of attention deficit/hyperactivity disorder

**DOI:** 10.1192/j.eurpsy.2025.30

**Published:** 2025-03-03

**Authors:** Annemette Hald, Ole Birger Pedersen, Kristoffer Sølvsten Burgdorf, Lise W. Thørner, Christina Mikkelsen, Lea A.N. Christoffersen, Henrik Ullum, Henrik Hjalgrim, Christian Erikstrup, Mie T. Bruun, Bitten Aagaard, Susan Mikkelsen, Thomas F. Hansen, Thomas Werge, Andrew J. Schork, Sisse Rye Ostrowski, Maria Didriksen

**Affiliations:** 1 Viro-Immunology Research Unit, Department of Infectious Diseases, Rigshospitalet Copenhagen University Hospital, Copenhagen, Denmark; 2 Department of Clinical Immunology, Zealand University Hospital, Køge, Denmark; 3 Novo Nordisk Foundation Center for Protein Research, University of Copenhagen, Copenhagen, Denmark; 4 Department of Clinical Immunology, Copenhagen University Hospital, Rigshospitalet, Copenhagen, Denmark; 5 Novo Nordisk Foundation Center for Basic Metabolic Research, Faculty of Health and Medical Science, Copenhagen University, Copenhagen, Denmark; 6 Institute of Biological Psychiatry, Copenhagen Mental Health Services, Copenhagen University Hospital, Roskilde, Denmark; 7 Statens Serum Institut, Copenhagen, Denmark; 8 Danish Cancer Society Research Center, Copenhagen, Denmark; 9 Department of Clinical Immunology, Aarhus University Hospital, Aarhus, Denmark; 10 Department of Clinical Medicine, Aarhus University, Aarhus, Denmark; 11 Department of Clinical Immunology, Odense University Hospital, Odense, Denmark; 12 Department of Clinical Immunology, Aalborg University Hospital, Aalborg, Denmark; 13 Department of Neurology, Danish Headache center/Danish Multiple Sclerosis Center, Copenhagen University Hospital, Rigshospitalet, Glostrup, Denmark; 14 Translational Research Centre, NeuroGenomic, Copenhagen University Hospital, Rigshospitalet Glostrup, Glostrup, Denmark; 15 Novo Nordisk Foundation Center for Protein Research, Copenhagen University, Copenhagen, Denmark; 16 Section for Geogenetics, GLOBE Institute, Faculty of Health and Medical Sciences, Copenhagen University, Copenhagen, Denmark; 17 Department of Clinical Medicine, Faculty of Health and Medical Sciences, University of Copenhagen, Copenhagen, Denmark; 18 Department of Neuroscience, Faculty of Health and Medical Sciences, University of Copenhagen, Copenhagen, Denmark

**Keywords:** ADHD presentation, ADHD symptomatology, ASRS, Depressive disorder, DBDS

## Abstract

**Background:**

Attention deficit/hyperactivity disorder (ADHD) prevalence has increased in the last 10 years, most likely due to increased recognition by clinicians. Even so, an issue with under-diagnostics may persist. Historically ADHD has been described as a male-dominant disorder. However, recent evidence shows that ADHD prevalence is similar between the sexes, but that the related impairment or symptomatology might vary. This study estimated the prevalence of undiagnosed ADHD symptoms (pADHD) and explored the sex-stratified symptomatology and associations with self-perceived health-related quality of life (HRQL) and experience of depressive symptoms.

**Methods:**

This was done in a unique cohort of 50,937 healthy blood donors – individuals who successfully maintain regular commitments despite potential ADHD symptoms. ADHD symptoms were estimated using the Adult ADHD Self-Report Scale (ASRS), health-related quality of life (HRQL) measured using mental and physical component scores (MCS/PCS) estimated based on a 12-item Short-Form Health Survey (SF-12) with a higher score indicating better HRQL, and depressive symptoms were measured using Major Depression Inventory (MDI) with higher score indicating more depressive symptoms.

**Results:**

In total, 3% were classified with pADHD (sex ratio 1:1). pADHD was associated with reduced MCS and PCS, and increased MDI score. Males scored on average higher on inattentive symptoms compared to females, whereas females scored on average higher on hyperactive-impulsive symptoms. Individuals scoring high on the combined inattentive and hyperactive-impulsive ADHD symptom presentation were most likely to be impaired in terms of higher MDI scores and lower PCS when compared to non-ADHD controls.

**Conclusions:**

In conclusion, ADHD symptoms are common in this seemingly healthy and undiagnosed population. Symptom presentations differ between sexes and the type of presentation seems to impact the association with depressive symptoms and level of reduced HRQL.

## Introduction

Attention-deficit/hyperactivity disorder (ADHD) affects between 1.5% and 3.6% of adults in European populations [1–3], representing a significant public health concern. Rather than being a binary condition, ADHD exists on a symptom continuum, with presentations varying across the lifespan as inattentive, hyperactive-impulsive, or combined [4]. The diagnosis requires onset of symptoms before age 12, and that an individual meets distinct deficits in core symptoms of inattention, hyperactivity, and impulsivity for at least six months. The symptoms must be occurring in two or more settings and must interfere with social or school functioning, and finally, the symptoms are not explained by another mental disorder [5]. The disorder often continues to impact multiple facets of life into adulthood, including impaired general functioning and well-being [6, 7].

ADHD symptoms are associated with significant distress due to feelings of depression and isolation, as well as self-esteem issues [6]. Beyond these direct negative impacts, individuals diagnosed with ADHD experience substantial psychosocial impairment due to high lifetime comorbidity rates of 60-80%, including a 45% lifetime prevalence of mood disorders [8, 9]. The more pronounced the diagnosed ADHD symptomatology, the more likely affected individuals will experience mental health comorbidity. Research indicates that having three or more psychiatric diagnoses is associated with a ten-fold increase in ADHD risk [1]. ADHD particularly impacts physical health through fatigue and energy depletion [6]. Individuals with diagnosed ADHD often experience exhaustion when controlling behavioral traits, as this requires substantial energy. These impacts can create a cycle of physical and mental health challenges that affect overall well-being [6].

Despite historically being described as a male-dominant, resulting in significant sex disparity in research, recent evidence shows similar ADHD prevalence between sexes, though the impact varies considerably [10]. The findings of Williamson and Johnston [11] suggest significant variation in ADHD comorbidity, psychosocial impairment, and cognitive functioning between sexes, supported by Faheem et al. [12]. Females more often receive a diagnosis in adulthood than males, possibly in part due to the disorder being masked by reductions in male hyperactive-impulsive symptom expression over time. This sex-based variation in presentation and diagnosis timing highlights the need for a more nuanced understanding of ADHD manifestation across populations.

While increased clinical recognition has improved diagnosis rates compared to prior decades, evidence suggests ADHD remains significantly underdiagnosed and undertreated in many European countries, particularly among individuals who have developed effective adaptive strategies [9, 10].

In Denmark, ADHD medication use increased by 71% in the last decade, especially among those aged 25-44, likely reflecting late diagnoses [13]. This trend suggests a substantial population of adults with unrecognized ADHD who may benefit from identification and support.

Current diagnostic practices may miss individuals who have developed successful coping mechanisms, particularly those who maintain regular employment and social commitments because related impairments may go beyond their abilities to uphold such. These individuals may experience significant symptoms and impairment while appearing to function well in daily life. Understanding this population’s experiences and needs requires novel research approaches that look beyond traditional clinical populations.

This study presents such an approach by examining the prevalence of ADHD symptoms in healthy adult blood donors – individuals who successfully maintain regular commitments despite potential symptoms. Unlike previous studies, which focused on clinical cases, our research specifically targets those who may have developed adaptive strategies to mask symptoms and remain undiagnosed. Blood donors represent a distinct study population as they must meet strict health criteria while maintaining regular employment and social commitments, potentially masking underlying ADHD symptoms through developed coping mechanisms.

Building on diagnostic criteria requiring onset before age 12, we hypothesize that undiagnosed and untreated ADHD symptoms are associated with poorer self-rated mental health and increased depressive symptoms later in life. Our study examines this relationship in a population that hasn’t sought clinical attention for these symptoms, potentially providing new insights into the long-term impact of undiagnosed ADHD.

## Methods

**Figure 1.**
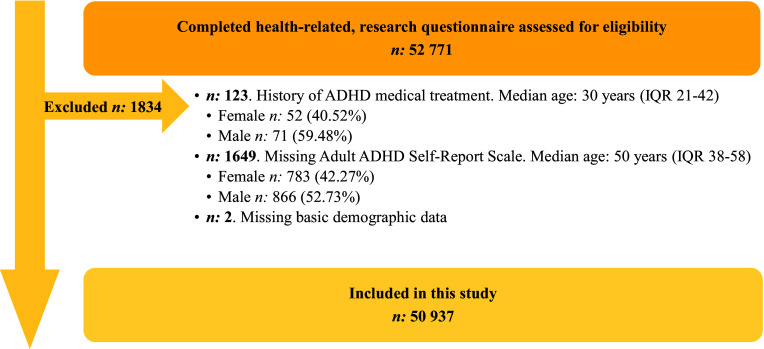
Displays inclusion of study participants.

The questionnaire was administered to 52,771 individuals. After filtering, 50,937 individuals were included in the study (Figure 1).

### Study population and material

This study utilized data from the Danish Blood Donor Study (DBDS), a national prospective cohort study and biobank established in 2010. The DBDS relies on existing Danish blood bank infrastructure, with donors invited to participate when visiting blood banks. Participants provide informed consent for individual-level information retrieval from Danish registers and complete web-based research questionnaires. Since participants are active blood donors upon inclusion, they meet general health requirements for donation eligibility [14, 15].

Data collection occurred between May 2015 and May 2018, including self-reported ADHD symptoms, lifestyle factors (height, weight, depression history, smoking status, alcohol habits), and demographic characteristics. Information on redeemed prescriptions and socio-economic characteristics came from Danish registers, including the Prescription Register and Population Register. After filtering, 50,937 individuals were included in the study. In this study the term “sex” refers to the sex the participants were assigned at birth, which was based solely on the visible external anatomy of a newborn.

#### Adult ADHD Self-Report Scale (ASRS)

The Adult ADHD Self-Report Scale V1.1 (ASRS), commonly used for adult ADHD screening and validated in general population samples [16], serves as a primary screening instrument in Danish clinical guidelines [17]. The scale contains 18 items scored on a five-point Likert scale (never to very often) based on six-month experiences. Nine items represent inattentive symptoms (items 1-4 and 7-11) and nine assess hyperactivity-impulsiveness (items 5–6 and 12–18). A total score ≥37 indicates probable ADHD, while subscale scores ≥24 identify specific presentations. It is possible to meet the criteria of ADHD (sum score ≥37) without scoring ≥24 in either or both subscales. For this study, we classified these individuals as non-specific. The ASRS has been reported to have good reliability and diagnostic utility among adults with a sensitivity of 0.92, a specificity of 0.69, and positive and negative predictive values of 0.48 and 0.97, respectively [18]. Similar psychometric properties were reported among Scandinavian adolescents [19].

#### Health-related quality of life (HRQL)

The 12-Item Short Form Health Survey (SF-12) assessed health-related quality of life through mental (MCS) and physical (PCS) components, validated across populations and particularly suitable for general population mental health measurement [20]. The scale comprises 12 items addressing four-week health state and health impacts on daily life. Component scores range from 0 to 100, with 100 representing optimal health status. Danish population means are 51 for PCS and 52.8 for MCS. Categories were defined following the Danish Health Authority methodology: the lowest 10% classified as “low,” the highest 65% as “good,” and the remaining 25% as “moderate” [21]. The SF-12 measures the following eight domains: General health perceptions, limitations in physical activities because of health problems, limitations in social activities because of physical or emotional problems, limitations in usual role activities because of physical health problems, bodily pain, general mental health (psychological distress and well-being), limitations in usual role activities because of emotional problems, and vitality (energy and fatigue).

#### Major Depression Inventory Scale (MDI)

The Major Depression Inventory Scale (MDI) measured current depression through ten symptoms within the previous two weeks, validated in Danish language [22]. The composite score ranges from 0 to 50, with scores above 20 indicating depression (21 for mild, 26 for moderate, 31 for severe depression). Additional questions assessed previous depression diagnosis and treatment history (medical or other).

### Statistics

The ADHD prevalence of ADHD symptoms (potential ADHD/pADHD) and the distribution of the individual characteristics and potential covariates are presented using descriptive statistics. Categorical and dichotomous variables are described in frequencies (*n*) and percentages (%) for females, males, and the total cohort comparing those with and without probable ADHD assessed according to the predefined full-edition cut-off. Initially, the normal distribution of continuous variables was tested by performing a Shapiro–Wilk normality test. Normally distributed variables were described by mean and standard deviation (SD), whereas non-normally distributed variables were described using the median and interquartile range (IQR). Differences in characteristics between pADHD and non-ADHD were investigated by using the Chi-squared test for dichotomous variables, by *t*-tests for normally distributed variables, and by Mann-Whitney *U*-tests for non-normally distributed variables. Also, the basic characteristics and frequencies of the specific pADHD presentations are illustrated along with measures of association (Risk Ratio). Difference in sex strata was tested by using Mantel-Haenszel, and results were presented as a Risk Ratio (MH RR) or as Female RR/Male RR when the Breslow-Day Test for Homogeneity was significant.

For analyzing the effect of pADHD (ASRS full edition) on the different assumed dependent mental health outcomes, crude multinomial logistic regression models were fitted for each outcome (scores of MDI and MCS, and history of depression).

Additional analyses were done to explore which factors impacted this association. For these, multivariate multinominal logistic regression models were computed. We explored the impact of age, history of depression, income level, educational level, employment status, number of children, BMI, alcohol consumption, and smoking status. Further, we included the investigated outcomes (depression, PCS, MCS) as covariates in models where they were not the outcome. The investigated covariates were selected based on previous literature. First, covariates were included in *fully* adjusted multinomial logistic regression analyses. Second, stepwise backward manual variable selection method was applied to the models to identify variables with any kind of impact on the associations between pADHD and MCS, PCS, and depression, respectively. The selected models are referred to as *final* models. Based on a priori knowledge these analyses were stratified by sex. All risk estimates (crude, fully adjusted, and final) are presented. Results are presented as odds ratios (OR) with a 95% confidence interval (CI), reported for each subgroup (sex and ADHD status). Based on the final multinomial logistic regression analyses, the indirect effects of each identified assumed mediator were calculated using the *nlcom* command in the statistical software Stata. We then determined the total indirect effect by summing the individual indirect effects. The proportion of the total indirect effect attributable to each mediator was calculated to understand the relative contribution of each mediator to the association between ADHD and outcomes of MCS and depression, respectively. Subsequently, sex-stratified Cox regression analyses were conducted to assess whether pADHD, and presentations of pADHD, predict later filled prescriptions for anti-depressive medication (ATC: N06A).

A test was considered statistically significant if the Bonferroni-corrected *p*-value <0.05, in multiple comparison analyses. All *p* values presented have been Bonferroni-corrected.

Analyses were performed using the SAS statistical software version 9.4 (SAS Institute, Cary, North Carolina, USA) and using Stata/SE version 18.0 (StataCorp, College Station, TX).

### Ethics

All participants have signed an informed consent form. Moreover, the study was registered in the Capital Region’s research directory (P-2019-99), and in the Scientific Research Ethics Committee system in the Central Denmark Region (1-10-72-95-13).

## Results

### Prevalence of ADHD symptoms

Characteristics of the study cohort are presented stratified by sex in Table 1. In 50 937 individuals, a total of 2.96% (*n*: 1507) had an ASRS score ≥37, which is henceforth classified as pADHD. Of these, 51.31% were male, (*p =* 0.04). Individuals with pADHD were on average younger than those without (*p* < 0.001). Except for BMI, there were significant differences between individuals with pADHD and those without on all other variables. A higher proportion of individuals with pADHD reported low HRQL and had a greater occurrence of depression both present and past. Additionally, they had a higher prevalence of unemployment, lower educational- and income level.

**Table 1.** Characteristics of the study population

		Female (*n*: 24 760)			Male (*n*: 26 177)		
		_p_ADHD	Non-_p_ADHD	*p*-Value	_p_ADHD	Non-_p_ADHD	*p*-Value
***n* (%)**		693 (2.8)	24 067 (97.2)	**<0.001**	814 (3.1)	25 363 (96.9)	**<0.001**
**ASRS score**							
**Median (IQR)**		40.0 (38.0–44.0)	19.0 (14.0–24.0)	**<0.001**	40.0 (38.0–45.0)	19.0 (14.0–24.0)	**<0.001**
**Age in years**							
**Median (IQR)**		27.9 (22.93–38.86)	39.3 (26.8–50.5)	**<0.001**	29.5 (25.3–38.2)	41.3 (30.1–51.5)	**<0.001**
**≤25**		269 (38.82)	4527 (18.2)	**<0.001**	192 (23.6)	3033 (11.9)	**<0.001**
**26–35**		196 (28.28)	5489 (22.2)		353 (43.4)	6046 (23.8)	
**36–45**		128 (18.47)	5023 (20.9)		146 (17.9)	5897 (23.3)	
**46–55**		71 (10.25)	5362 (22.3)		97 (11.9)	6063 (23.9)	
**>55**		29 (4.18)	3666 (15.2)		26 (3.2)	4324 (17.1)	
**Perception of health (SF–12)**							
*Mental component summary*							
**MCS score median (IQR)**		39.9 (29.4–48.8)	53.2 (47.5–56.7)	**<0.001**	44.8 (34.9–51.2)	54.7 (49.6–56.7)	**<0.001**
**Low MCS**		337 (48.6)	2675 (11.1)	**<0.001**	278 (34.1)	1715 (6.8)	**<0.001**
**Moderate MCS**		215 (31.0)	6181 (25.7)		305 (37.5)	5778 (22.8)	
**Good MCS**		127 (18.3)	14626 (60.8)		216 (26.5)	17286 (68.2)	
***Missing***		14 (2.0)	585 (2.4)		15 (1.8)	584 (2.3)	
*Physical component summary*							
**PCS score median (IQR)**		55.6 (51.1–58.4)	55.5 (53.1–56.9)	0.5426	55.6 (51.6–57.9)	55.5 (53.2–56.8)	0.4838
**Low PCS**		141 (20.4)	2475 (10.3)	**<0.001**	137 (16.8)	2175 (8.6)	**<0.001**
**Moderate PCS**		148 (21.4)	5611 (23.3)		192 (23.6)	6433 (25.4)	
**Good PCS**		390 (56.3)	15396 (63.9)		470 (57.7)	16171 (63.8)	
***Missing***		14 (2.0)	585 (2.4)		15 (1.8)	584 (2.3)	
**Presence of depressive symptoms**							
**MDI score median (IQR):**		14.0 (9.0–22.0)	4.0 (2.0–7.0)	**<0.001**	12.0 (7.0–19.0)	4.0 (1.0–7.0)	**<0.001**
**No depressive disorder**		476 (68.7)	23068 (95.9)	**<0.001**	640 (78.6)	24649 (97.2)	**<0.001**
**Mild depressive disorder**		87 (12.6)	374 (1.6)		68 (8.4)	257 (1.1)	
**Moderate depressive disorder** **Severe depressive disorder**		62 (8.9)	222 (0.9)		62 (7.6)	117 (0.5)	
		61 (8.8)	143 (0.6)		33 (4.1)	68 (0.3)	
**Present depressive disorder**		210 (30.3)	739 (3.1)	**<0.001**	163 (20.0)	442 (1.7)	**<0.001**
***Missing***		7 (1.1)	260 (1.1)		11 (1.4)	272 (1.1)	
**History of diagnosed depression**							
**No**		497 (71.7)	21506 (89.4)	**<0.001**	699 (85.9)	23910 (94.3)	**<0.001**
**Yes**		195 (28.1)	2518 (10.5)		114 (14.0)	1406 (5.5)	
***Missing***		-	43 (0.2)		-	47 (0.2)	
Received medical treatment^*^							
Self-reported yes		89 (45.6)	1253 (49.8)	**<0.001**	50 (43.9)	711 (50.6)	**<0.001**
Self-reported no		106 (54.4)	1262 (50.1)		64 (56.1)	688 (48.9)	
***Missing***		0	-		0	7 (0.1)	
**Anti-depressive medication in prescription register**		89 (12.8)	1,253 (5.9)		50 (6.1)	711 (2.8)	**<0.001**
**Received other treatment**		134 (68.7)	1782 (70.8)	**<0.001**	87 (76.3)	924 (65.7)	**<0.001**
**No**		61 (31.3)	729 (28.9)		27 (23.7)	480 (34.1)	
***Missing***		0	7 (0.3)		0	-	
**The highest achieved educational level**							
**Compulsory**		164 (23.7)	2938 (12.2)	**<0.001**	127 (15.6)	2844 (11.2)	**<0.001**
**Upper secondary/vocational/other**		222 (32.0)	7430 (30.9)		302 (37.1)	11226 (44.3)	
**Diploma/bachelor**		214 (30.9)	9636 (40.0)		250 (30.7)	7494 (29.6)	
**Master or PhD**		92 (13.3)	3891 (16.2)		125 (15.4)	3571 (14.1)	
***Missing***		-	172 (0.7)		10 (1.2)	288 (0.9)	
**Employment status**							
**Employed**		516 (74.5)	20277 (84.3)	**<0.001**	692 (85.0)	22646 (89.3)	**<0.001**
**Unemployed**		23 (3.3)	463 (1.9)		18 (2.2)	436 (1.7)	
**Student/retired**		154 (22.2)	3327 (13.8)		104 (12.8)	2281 (8.9)	
**Income level**							
**Low**		268 (38.7)	5343 (22.2)	**<0.001**	196 (24.1)	4256 (16.8)	**<0.001**
**Low-medium**		192 (30.2)	6223 (25.9)		209 (23.6)	3560 (14.4)	
**Medium**		103 (14.9)	5432 (22.6)		158 (19.4)	4474 (17.6)	
**High-medium** **High**		74 (10.7)	4406 (18.3)		135 (16.6)	5629 (22.9)	
		39 (5.6)	2663 (11.1)		133 (16.3)	7444 (29.4)	
**Number of children**							
**0**		437 (63.1)	13237 (55.0)	**0.0029**	477 (58.6)	11869 (46.8)	**<0.001**
**1**		97 (14.0)	3596 (14.9)		137 (16.8)	4351 (17.2)	
**2**		111 (16.0)	4880 (20.3)		142 (17.4)	5908 (23.3)	
**≥3**		42 (6.1)	1584 (6.6)		56 (6.9)	2245 (8.9)	
***Missing***		6 (0.9)	770 (3.2)		-	990 (3.9)	
**Body mass index**							
**Score median (IQR)**		24.6 (22.4–28.0)	24.4 (22.2–27.6)	0.0809	25.5 (23.4–27.8)	25.5 (22.8–27.9)	0.2449
**Underweight**		6 (0.9)	6 (0.9)	0.1554	-	75 (0.3)	0.6377
**Normal weight**		363 (52.4)	13295 (55.2)		361 (44.4)	11009 (43.4)	
**Overweight**		206 (29.7)	7025 (29.2)		333 (40.9)	11050 (43.6)	
**Moderately obese**		88 (12.7)	2455 (10.2)		91 (11.2)	2529 (9.9)	
**Severely obese**		16 (2.3)	754 (3.1)		19 (2.3)	527 (2.1)	
**Morbidly obese**		11 (1.6)	277 (1.2)		6 (0.7)	140 (0.6)	
***Missing***		-	90 (0.4)		-	33 (0.1)	
**Frequency of alcohol consumption**							
**Never/almost never**		367 (52.9)	15251 (63.4)	**<0.001**	388 (47.7)	13855 (54.6)	**<0.001**
**Sometimes a month**		291 (41.9)	8056 (33.5)		366 (44.9)	10196 (40.2)	
**Sometimes a week**		31 (4.5)	375 (1.6)		46 (5.7)	999 (3.9)	
**Daily/almost daily**		-	13 (0.1)		5 (0.6)	51 (0.2)	
***Missing***		-	372 (1.6)		9 (1.1)	262 (1.0)	
**Current smoking status**							
**Non-smoker**		503 (72.6)	20888 (86.7)	**<0.001**	650 (79.9)	22106 (87.2)	**<0.001**
**Smoker**		187 (26.9)	3132 (13.0)		161 (19.8)	3207 (12.6)	
***Missing***		-	47 (0.2)		-	50 (0.2)	

Note. *n*: number. IQR: Interquartile range. Level of significance: *p*-value < 0.05 (in **bold**). “-”: less than five individuals (according to the terms and conditions of Danish legislation (the Danish Act on Processing of Personal Data) tables must contain at least 5 units per cell).

_p_ADHD: Possible ADHD; ASRS: WHO Adult ADHD Self-Report Scale. ADHD is considered present with an ASRS full edition score equal to or above 37.

MDI: Major Depression Inventory Scale. Mild depression = MDI score of 21, moderate depression = MDI score of 26. Severe depression = MDI score of 31. Present depressive disorder = MDI score equal to or above 21.

SF-12: 12-Item Short Form Health Survey. PCS: Physical Component Summary. MCS: Mental Component Summary. Low = the 10% of the population scoring the lowest. Good = the 65% of the population scoring the best. Moderate = the remaining 25%.

BMI: Body Mass Index (Underweight: BMI <18.5) (Normal weight: BMI 18.5-24.9) (Overweight: BMI 25.0-29.9) (Moderately Obese: BMI 30.0-34.9) (Severely Obese: BMI 35.0 39.9) (Morbidly Obese: BMI ≥40.0).

* Items on self-reported depression treatment was only asked to those who reported having a history of diagnosed depression.

#### Possible ADHD presentation

**Figure 2.**
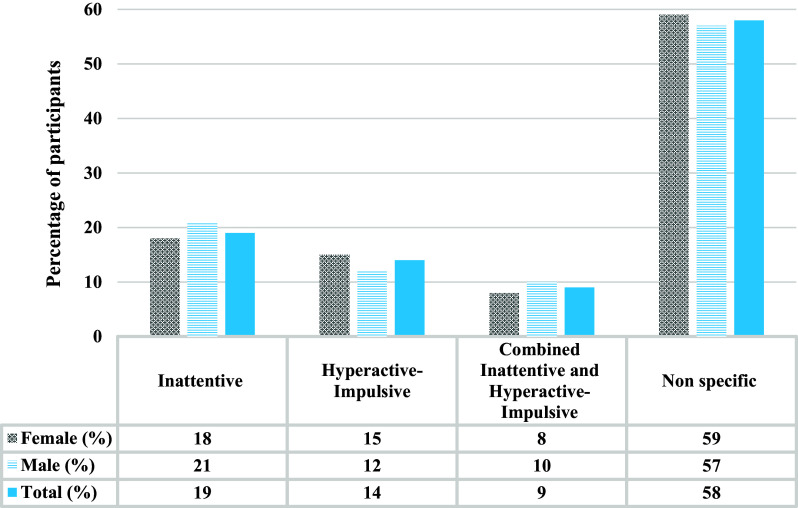
Displays the distribution of individuals characterised with the different ADHD presentations.

Of the 1,507 individuals who were classified with pADHD, the non-specific presentation was the most common (Figure 2).

The average MDI scores varied according to ASRS score in both females and males with the highest MDI score observed for the group of individuals classifying with the combined inattentive and hyperactive-impulsive pADHD presentation. The average MCS only varied significantly according to ASRS score in males where the lowest average MCS was observed for the inattentive pADHD presentation (Table 2).

**Table 2.** Mental and physical health scores (MDI and SF-12) of individuals classified with possible ADHD according to the full ASRS, and the specific ADHD presentations

Scoring method	ASRS full edition	Inattentive subscale	Hyperactive-impulsive subscale	Combined inattentive and hyperactive-impulsive subscale	Non-specific presentation	
**Cut-off**	≥37	≥24	≥24	≥24/≥24	≥37	**P value**
**ASRS items**	1–18	1–4+7–11	5–6+12–18	1–4+7–11/5–6+12–18	1–18	
**ASRS score, median (IQR)**	40.0 (38.0–44.0)	43.0 (40.0–47.0)	43.0 (39.0–46.0)	54.0 (51.0–58.0)	39.0 (38.0–40.0)	<0.001
Female	40.0(38.0–44.0)	44.0 (39.0–47.0)	42.0 (39.0–46.0)	56.0 (52.0–58.0)	39.0 (38.0–41.0)	<0.001
Male	40.00 (38.0–45.0)	43.0 (39.0–47.0)	43.0 (40.0–47.0)	54.0 (51.0–56.0)	39.0 (38.0–40.0)	
***n* (%)**	1507 (100.0)	289 (19.2)	202 (13.4)	136 (9.0)	880 (58.4)	
Female	693 (48.7)	121 (41.9)	102 (50.5)	58 (42.7)	412 (46.8)	0.22
Male	814 (51.3)	168 (58.1)	100 (49.5)	78 (57.4)	468 (53.2)	0.02
**Age in years, *n* (%)**						0.01
≤25	461 (30.6)	103 (35.6)	49 (24.3)	45 (33.1)	264 (30.0)	
26–35	549 (36.4)	102 (35.3)	80 (39.6)	57 (41.9)	310 (35.2)	
36–45	274 (18.2)	48 (16.6)	33 (16.3)	20 (14.7)	173 (19.7)	
46–55	168 (11.2)	32 (11.1)	32 (15.8)	10 (7.4)	94 (10.7)	
>55	55 (3.7)	4 (1.4)	8 (3.9)	4 (2.9)	39 (4.4)	
**MDI score median (IQR)**						<0.001
Female	14.0 (9.0–22.0)	16.00 (8.0–24.0)	13.00 (8.0–20.0)	20.00 (11.0–30.0)	14.00 (9.00–21.00)	<0.001
Male	12.0 (7.0–19.0)	14.00 (9.0–22.0)	11.00 (7.0–17.0)	18.00 (9.0–25.0)	11.00 (7.00–16.00)	0.01
**MCS score median (IQR)**						<0.001
Female	39.9 (29.4–48.8)	37.7 (26.1–47.7)	42.9 (33.9–49.9)	35.8 (24.4–50.7)	40.0 (30.2–48.9)	0.11
Male	44.8 (34.9–51.2)	40.2 (32.2–48.1)	48.7 (38.0–54.3)	42.9 (28.7–49.9)	45.7 (37.5–51.3)	<0.001
**PCS score median (IQR)**						0.01
Female	55.6 (51.1–58.4)	56.1 (51.1–58.9)	56.8 (52.9–58.8)	54.4 (47.5–59.0)	55.1 (51.0–58.0)	0.14
Male	55.6 (51.6–57.9)	55.2 (50.80–6.8)	56.2 (53.1–58.4)	54.9 (51.3–58.1)	55.5 (51.7–57.8)	0.06

Note. *n*: number. IQR: Interquartile range.

*p* Value was estimated using Kruskall–Wallis test.

ASRS: WHO Adult ADHD Self-Report Scale ASRS full edition was assessed using questions 1 through 18 score and ADHD is considered present with a score equal to or above 37. Different presentations of ADHD (in participants with full edition score ≥37) were considered using different blocks (items) of questions from the ASRS:

- The inattentive ADHD subscale was assessed using questions 1 through 4 and 7 through 11 (inattentive symptoms are considered present with a score equal to or above 24).

- The hyperactivity-impulsivity ADHD subscale was assessed using questions 5 through 6 and 12 through 18 (hyperactivity-impulsivity symptoms are considered present with a score equal to or above 24).

- The combined Inattentive and Hyperactive-Impulsive were assessed using questions 1 through 18 (combined symptoms are considered present with a score equal to or above 24 on both subscales).

- The non-specific presentation was assessed using questions 1 through 18 (non-specific symptoms are considered present with a total score above or equal to 37 but below 24 in either subscale).

SF-12: 12-Item Short Form Health Survey. PCS: Physical Component Summary. MCS: Mental Component Summary.

MDI: Major Depression Inventory Scale. MDI score equal to or above 21 = depressive disorder.

When adjusting for multiple comparisons using Bonferroni correction there were no statistically significant variations in risk of presenting with a specific set of ADHD symptoms depending on sex. Females with pADHD had a nominally significant 24% lower risk (non-adjusted *p =* 0.01) of having an inattentive presentation compared to males with pADHD (Table 3).

**Table 3.** Risk ratios (RR) and 95% confidence intervals (Cl) of the association between the different presentations and sex among possible ADHD participants

	RR (95% Cl)	*p*-Value
**Inattentive**	0.76 (0.6–0.9)	0.0572
**Hyperactive-impulsive**	1.04 (0.8–1.3)	1.0
**Combined inattentive and hyperactive-impulsive**	0.88 (0.6–1.1)	1.0
**Non-specific**	0.93 (0.8–1.1)	1.0

Note. Comparison group: females with possbile ADHD vs. males with possible ADHD. A Bonferonni-corrected *p*-value < 0.05 is considered statistically significant.

### Depressive symptoms and health-related quality of life

Irrespective of the ASRS scoring method (total score or only using items representing each specific presentation), experiencing pADHD symptoms was associated with a 3.5–5 times higher risk of having a low mental health component score as well as a 2–4 times higher risk of having been diagnosed with a depression in the past in both females and males (Table 4). The combined inattentive and hyperactive-impulsive pADHD presentation had the highest prevalence of both former and present depression, reflected in a 17.5 times higher risk (crude RR 17.48, 95%-Cl 11.2–21.5) of co-occurring depressive symptoms compared to the non-ADHD group.

**Table 4.** Frequency and associations (RR) of mental and physical health-related quality of life, and depression, respectively with ADHD

Scoring method	ASRS full edition				
**Cutoff**	≥37				
**ASRS items**	1–18				
	***n* (%)**	**Crude RR (95% Cl)**	***p*-Value**	**MH RR (95% Cl)**	***p*-Value**
**Perception of health (SF–12)**					
Low mental health state (MCS)	612 (41.41)	4.6 (4.3–4.9)	**<0.01**	4.6 (4.3–5.0)	**<0.01**
Moderate/Good mental health state (MCS)	866 (58.59)	1.0		1.0	
Low physical health state (PCS)	282 (19.08)	1.9 (1.8–2.2)	**<0.01**	2.0 (1.8–2.2)	**<0.01**
Moderate/Good physical health state (PCS)	1196 (80.92)	1.0		1.0	
**Present depressive disorder**					
Yes	373 (25.05)	10.4 (9.3–11.5)	**<0.01**	10.5 (9.5–11.7)	**<0.01**
No	1116 (74.95)	1.0		1.0	
**History of diagnosed depression**					
Yes	309 (20.53)	2.6 (2.3–2.9)	**<0.01**	2.6 (2.4–2.9)	**<0.01**
No	1196 (79.47)	1.0		1.0	

**Scoring method**	**Inattentive subscale**				
**Cut-off**	≥24				
**ASRS items**	1–4+7–11				
	***n* (%)**	**Crude RR (95% Cl)**	***p*-Value**	**MH RR (95% Cl)**	***p*-Value**
**Perception of health (SF–12)**					
Low mental health state (MCS)	144 (50.70)	5.2 (4.6–5.8)	**<0.01**	5.2 (4.7–5.9)	**<0.01**
Moderate/Good mental health state (MCS)	140 (49.30)	1.0		1.0	
Low physical health state (PCS)	58 (20.42)	2.1 (1.6–2.6)	**<0.01**	2.0 (1.6–2.5)	**<0.01**
Moderate/Good physical health state (PCS)	226 (79.58)	1.0		1.0	
**Present depressive disorder**					
Yes	96 (33.57)	11.5 (9.7–13.7)	**<0.01**	11.9 (10.1–14.0)	**<0.01**
No	190 (66.43)	1.0		1.0	
**History of diagnosed depression**					
Yes	68 (22.01)	2.9 (2.3–3.5)	**<0.01**	3.0 (2.4–3.6)	**<0.01**
No	221 (77.90)	1.0		1.0	

**Scoring method**	**Hyperactive-impulsive subscale**				
**Cut-off**	≥24				
**ASRS items**	5–6+12–18				
	***n* (%)**	**Crude RR (95% Cl)**	***p*-Value**	**MH RR (95% Cl)**	***p*-Value**
**Perception of health (SF–12)**					
Low mental health state (MCS)	72 (36.36)	3.7 (3.1–4)	**<0.01**	3.5 (2.9–4.2)	**<0.01**
Moderate/Good mental health state (MCS)	126 (63.64)	1.0		1.0	
Low physical health state (PCS)	30 (15.15)	1.5 (1.1–2.1)	0.062	1.5 (1–1–2–0)	0.073
Moderate/Good physical health state (PCS)	168 (84.85)	1.0		1.0	
**Present depressive disorder**					
Yes	42 (21.11)	7.0 (5.3–9.2)	**<0.01**	6.5 (5.0–8.5)	**<0.01**
No	157 (78.89)	1.0		1.0	
**History of diagnosed depression**					
Yes	36 (17.82)	2.2 (1.6–2.9)	**<0.01**	2.2 (1.6–2.8)	**<0.01**
No	166 (82.18)	1.0		1.0	

**Scoring method**	**Combined inattentive and hyperactive-impulsive subscale**								
**Cut-off**	≥24/≥24								
**ASRS items**	1–4+7–11/5–6+12–18								
	***n* (%)**	**Crude RR (95% Cl)**	***p*-Value**	**MH RR (95% Cl)**	***p*-Value**	**Female RR (95% Cl)**	***p*-Value**	**Male RR (95% Cl)**	***p*-Value**
**Perception of health (SF–12)**									
Low mental health state (MCS)	63 (46.67)	5.2 (4.3–6.2)	**<0.01**	4.9 (4.1–5.8)	**<0.01**				
Moderate/Good mental health state (MCS)	72 (53.33)	1.0		1.0					
Low physical health state (PCS)	33 (24.44)	2.5 (1.9–3.4)	**<0.01**	2.5 (1.8–3.3)	**<0.01**				
Moderate/Good physical health state (PCS)	102 (75.56)	1.0		1.0					
**Present depressive disorder**									
Yes	57 (42.22)	17.48 (11.2–21.5)	**<0.01**	14.8 (12.0–17.9)	**<0.01**				
No	78 (57.78)	1.0		1.0					
**History of diagnosed depression**									
Yes	37 (27.21)	3.4 (2.6–4.5)	**<0.01**			4.1 (3.1–5.5)	**<0.01**	2.4 (1.4–4.2)	**<0.01**
No	99 (72.79)	1.0				1.0		1.0	

**Scoring method**	**Non-specific presentation**				
**Cut-off**	≥37				
**ASRS items**	1–18				
	***n* (%)**	**Crude RR (95% Cl)**	***p*-Value**	**MH RR (95% Cl)**	***p*-Value**
**Perception of health (SF–12)**					
Low mental health state (MCS)	333 (54.41)	4.3 (3.9–4.7)	**0.01**	4.1 (3.8–4.5)	**<0.01**
Moderate/Good mental health state (MCS)	528 (8.31)	1.0		1.0	
Low physical health state (PCS)	161 (18.70)	1.9 (1.7–2.2)	0.715	1.9 (1.7–2.2)	**<0.01**
Moderate/Good physical health state (PCS)	700 (81.30)	1.0		1.0	
**Present depressive disorder**					
Yes	178 (47.72)	8.5 (7.4–9.8)	**<0.01**	7.4 (6.5–8.5)	**<0.01**
No	691 (61.92)	1.0		1.0	
**History of diagnosed depression**					
Yes	168 (19.13)	2.4 (2.1–2.8)	0.256	2.4 (2.1–2.7)	**<0.01**
No	710 (80.87)	1.0		1.0	

*Note.* Comparison: ADHD (screening positive on the specific subscale) vs. Non-ADHD (ASRS score <37). *n*: number. p-Value (level of significance: Bonferroni-corrected *p*-value< 0.05 in **bold**).

Mantel–Haenszel Risk Ratio (MH RR): adjusted for sex, female/male RR present sex-stratified estimates when Breslow–Day test for Homogeneity was significant.

ASRS: WHO Adult ADHD Self-Report Scale. ASRS full edition was assessed using questions 1 through 18 score and ADHD is considered present with a score ≥37. Different presentations of ADHD were considered using different blocks (items) of questions from the ASRS:

- The inattentive ADHD subscale was assessed using questions 1 through 4 and 7 through 11 (inattentive symptoms are considered present with a score ≥24).

- The hyperactivity-impulsivity ADHD subscale was assessed using questions 5 through 6 and 12 through 18 (hyperactivity-impulsivity symptoms are considered present with a score ≥24)

- The combined Inattentive and Hyperactive-Impulsive were assessed using questions 1 through 18 (combined symptoms are considered present with a score ≥24 on both subscales).

- The non-specific presentation was assessed using questions 1 through 18 (non-specific symptoms are considered present with a total score above or equal to 37 but below 24 in either subscale).

Present depressive disorder = Major Depression Inventory Scale score ≥21.

SF-12: 12-Item Short Form Health Survey. PCS: Physical Component Summary. MCS: Mental Component Summary. Low = the 10% of the population scoring the lowest. Moderate/Good = the remaining 90%.

Similar propensities regarding lower HRQL and the occurrence of depressive symptoms in participants with pADHD were observed in the sex-stratified regression analysis (Table 5). The odds for a low MCS score were 13.6 (95% CI: 11.1–16.7) and 12.6 (95% CI:10.5–15.1) times higher in females and males with pADHD symptoms compared to those without. The associations remained statistically significant after adjustments, but the risk estimates were reduced. A similar pattern was observed for the MDI-score.

**Table 5.** Association between possible ADHD and mental health characteristics based on multinominal logistic regression analyses including possible ADHD as the independent variable and the mental health outcomes as dependent variables

	Female (*n: 24 760*)						Male (*n: 26 177*)					
Outcome	Crude OR (95% CI)	*p*-Value	Fully adjusted OR (95% CI)	*p*-Value	Final OR (95% CI)	*p*-Value	Crude OR(95% CI)	*p*-Value	Fully adjusted OR (95% CI)	*p*-Value	Final OR (95% CI)	*p*-Value
**MCS score**												
Low MCS	13.6 (11.1–16.7)	**<0.001**	5.8 (4.6–7.4)	**<0.001**	6.9 (5.5–8.7)	**<0.001**	12.6 (10.5–15.1)	**<0.001**	5.8 (4.7–7.3)	**<0.001**	56.8 (5.5–8.4)	**<0.001**
Moderate MCS	3.8 3.0–4.7)	**<0.001**	2.8 (2.1–3.6)	**<0.001**	3.2 (2.6–3.9)	**<0.001**	4.01 (3.5–4.9)	**<0.001**	2.9 (2.4–3.5)	**<0.001**	3.5 (2.9–4.2)	**<0.001**
Good MCS	1.0		1.0		1.0		1.0		1.0		1.0	
**MDI score**												
No depressive disorder	1.0		1.0		1.0		1.0		1.0		1.0	
Mild depressive disorder	11.3 (8.7–14.5)	**<0.001**	3.3 (2.4–4.3)	**<0.001**	3.5 (2.6–4.6)	**<0.001**	10.2 (7.7–13.5)	**<0.001**	2.7 (1.9–3.7)	**<0.001**	2.9 (2.1–4.0)	**<0.001**
Moderate depressive disorder	13.5 (10.0–18.2)	**<0.001**	3.7 (2.6–5.1)	**<0.001**	4.1 (2.9–5.7)	**<0.001**	20.4 (14.9–28.0)	**<0.001**	5.1 (3.6–7.3)	**<0.001**	5.4 (3.8–7.7)	**<0.001**
Severe depressive disorder	20.7 (15.1–28.3)	**<0.001**	4.8 (3.4–6.9)	**<0.001**	5.5 (3.9–7.8)	**<0.001**	18.7 (12.2–28.5)	**<0.001**	4.9 (3.1–7.9)	**<0.001**	5.2 (3.3–8.1)	**<0.001**

Note. Comparison groups: possible ADHD vs. Non-ADHD (with complete data: “n:”). ADHD is considered present with a WHO adult ADHD Self-Report Scale full edition score equal to or above 37. *n*: number. *p*-Value (level of significance: < 0.05 in **bold**).

MDI: Major Depression Inventory Scale. No depressive disorder = MDI score equal to or less than 20. Mild depression = MDI score >21<26, moderate depression = MDI score >26<31. Severe depression = MDI score >31. Present depressive disorder = MDI score equal to or above 21.

SF-12: 12-Item Short Form Health Survey. MCS: Mental Component Summary. Low = the 10% of the population scoring the lowest. Good = the 65% of the population scoring the best. Moderate = the remaining 25%.

Models:

Crude: unadjusted Fully adjusted: Adjusted for age (continuous) and all other covariates except the specific outcome in question (history of depression, body mass index (BMI), alcohol consumption ("Sometimes a week" coupled with “Daily/almost daily” due to low prevalence of daily drinkers), smoking status, income level, educational level, employment status, number of children, and mental/physical health wherever this was not included as the dependent variable. Final: Adjusted for age (continuous), and variables that remained statistically associated with the outcome variable at the level of *p* < 0.05 after backward selection:
MCS outcome:
*Female:* number of children, PCS score, MDI score, History of depression, BMI, Smoking status
*Male:* number of children, Employment status, educational level, Income level, PCS score, MDI score, History of depression, BMI, Smoking status MDI outcome:
*Female:* number of children, Employment status, MCS score, PCS score, History of depression, Smoking status
*Male:* number of children, Income level, MCS score, PCS score, History of depression, BMI

### Mediation tests

Number of children, general physical health, and previous depression impacted the observed associations for both sexes. In males, it appeared that socioeconomic status had a bigger impact compared to females.

The covariates identified in the model search were included in mediation tests to estimate the proportion of the total indirect effect attributable to each of these.

It was observed that 14% of the total association between pADHD and MDI score among females was explained by the pathways going through all the included mediators combined, while 8.9% was explained among males. In both sexes, most of the estimated indirect effect between pADHD and MDI score was explained by MCS score (90.4% for females and 93.1% for males), whereas 4.0% was explained by history of depression for females, and 4.9% was explained by registered income level for males. Also, it was observed that 35% of the association between pADHD and MCS score was explained by pathways going through all included mediators in females, while this was 24.4% in males. For this association, the main mediator pathway was through MDI score (89.6%% for females and 84.4% for males), whereas 5.9% was explained by history of depression for females, and 8.6% was explained by registered income level for males.

The isolated indirect effects as well as the proportion of the total indirect effect explained by each of the mediators are displayed in Table 6.

**Table 6.** Displaying indirect effects explained by each mediator pathway

Mediator	Female (n: 24,760)						Male (n: 26,177)					
	MCS score			MDI score			MCS score			MDI score		
	Coef.	*p* Value	%^×^	Coef.	*p* Value	%^×^	Coef.	*p* Value	%^×^	Coef.	*p* Value	%^×^
Total indirect effect^*^	–0.347 (–0.368- –0.326)	<0.001	35	0.144 (0.134–0.154)	<0.001	14	−0.244 (− 0.259 to −0.228)	<0.001	24.4	0.089 (0.083–0.095)	<0.001	8.9
Number of children	–0.002 (–0.004- –0.001)	0.011	0.6	0.001 (0.000–0.002)	0.01	0.7	0.001 (–0.00 to 0.003)	0.140	0	0.001 (0.000–0.002)	0.005	1.2
PCS score	–0.002 (–0.004–0.000)	0.118	0.5	0.001 (0.000–0.003)	0.023	1.0	−0.006 (−0.009 to −0.004)	<0.001	2.6	−0.001 (−0.00 to0.00)	0.104	0
MCS score				0.130 (0.121–0.139)	<0.001	90.4				0.083 (0.077 to −0.089)	<0.001	93.1
MDI score	–0.311 (–0.331- –0.291)	<0.001	89.6				−0.206 (−0.220 to −0.191)	<0.001	84.4			
History of depression	–0.021 (–0.026- –0.015)	<0.001	5.9	0.006 (0.004–0.008)	<0.001	4.0	−0.007 (−0.010 to –0.004)	<0.001	3.0	0.001 (0.000–0.002)	0.002	1.4
Body mass index	0.000 (–0.000 – 0.001)	0.905	0	-	-	-	0.000 (−0.000–0.000)	0.883	0	−0.000 (−0.000 to 0.000)	0.637	0
Smoking status	–0.011 (–0.015- –0.007)	<0.001	3.3	0.002 (0.000–0.004)	0.038	1.4	−0.004 (−0.006 to −0.002)	<0.001	1.5	-	-	-
Employment status	-	-	-	0.003 (0.002–0.004)	<0.001	2.3	−0.001 (−0.003 to 0.000)	0.084	0.5	-	-	-
Income level	-	-	-	-	-	-	−0.211 (−0.026 to −0.016)	<0.001	8.6	0.004 (0.077–0.089)	<0.001	4.9

Note. Coef. = coefficient (95% confidence interval).

* The total indirect effect explains how much of the association between pADHD and the outcome in question is explained by pathways through the included mediators collectively.

× Proportion of the total indirect effect explained by the mediator.

### ADHD symptoms and subsequent anti-depressive treatment

Overall, 1934 participants received a prescription of anti-depressive medication after inclusion date (median number of days after inclusion = 1485, interquartile range (IQR): 891–1892). In total, 652 (14.9%) of these reported having a previous depression diagnosis. For the Cox regression analysis, the date of censoring was set as the date of filled prescription, date of death, or December 31^st^, 2022, whichever came first. Each participant was observed at risk of being prescribed anti-depressive medicine for 5.9 person-years on average (a total of 311,419.75 person-years). The hazard of being prescribed anti-depressive medication after the ADHD assessment was increased for those with pADHD compared to those without ADHD symptoms in both sexes. The inattentive and combined presentations were associated with the most increased hazard in both sexes (Table 7).

**Table 7.** ADHD symptomatology and risk of subsequently being prescribed anti-depressive medication. Cox regression analysis

	Men (*n* = 26,177)		Women (*n*= 24,760)	
ADHD symptomatology	HR (95% CI)	*p* Value	HR (95% CI)	*p* Value
Possible ADHD	3.34 (2.62–4.26)	<0.01	3.58 (2.91–4.41)	<0.01
Hyperactive-impulsive	3.04 (1.51–6.10)	0.012	3.49 (2.06–5.91)	<0.01
Inattentive	5.25 (3.47–7.96)	<0.01	4.79 (3.14–7.31)	<0.01
Combined inattentive and hyperactive-impulsive	3.47 (1.65–7.31)	0.006	6.02 (3.48–10.4)	<0.01
Non-specific	2.71 (1.92–3.82)	<0.01	2.94 (2.20–3.93)	<0.01

## Discussion

Our study reveals several important findings regarding undiagnosed ADHD in well-functioning adults. In this healthy cohort meeting strict blood donation criteria, we found substantial pADHD prevalence (~3%) with significant impacts on health-related quality of life and depression risk. These findings in individuals who successfully navigated life’s demands without formal diagnosis suggest ADHD symptoms may persist and impact well-being even in seemingly well-adjusted adults.

The prevalence of pADHD reported here is similar to what has been estimated in populations throughout developed and developing countries. In childhood, ADHD is among the most common psychiatric disorders with a prevalence rate of 3–5% [9], and 5.9% of youth [10]. A 2020 systematic review and meta-analysis including older adults reported a prevalence ranging from 1.5% to 2.2% [3]. This is on the lower end compared to estimates reported in a 2017 meta-analysis, based on the WHO World Mental Health Surveys across 20 countries, which found an ADHD prevalence of 3.6% in high-income countries [1]. The differences in the prevalence of adult ADHD across studies could suggest variations in the expression of ADHD related to developmental change, cultural differences across populations, and/or due to differences in methodology across studies [23]. Prevalence estimates strongly depend on diagnostic tools used (e.g., ICD-10 vs. DSM-IV or DSM-5), informant type (such as parent/teacher vs. self-reports). This could also be speculated to be due to underestimation of the true number of ADHD cases or the occurrence of a late-onset ADHD syndrome [24]. Moreover, our findings support previous evidence of the combined or non-specific presentation of ADHD being the most prevalent (estimated 50–75% of cases) [25]. However, it has been reported that in adult populations, the inattentive subtype predominates as hyperactive-impulsive symptoms attenuate, often manifesting with functional impairments that may be misinterpreted as mood or anxiety disorders [26].

A key aspect of our study is the examination of ADHD-depression relationships in undiagnosed individuals. While the correlation between diagnosed ADHD and Major Depressive Disorder (MDD) is well-documented [27] with lifetime MDD prevalence ranging from 11 to 50% among adults with ADHD [8, 28], the impact of undiagnosed ADHD symptoms on depression risk has remained largely unexplored. Our findings reveal that 25% of participants with pADHD reported current depressive symptoms, with an increased hazard of anti-depressant medication prescription up to 5 years after assessment.

Particularly noteworthy was our finding that approximately 20% of individuals with pADHD reported prior depression diagnosis while remaining untreated for ADHD. This observation may suggest a potentially critical gap in clinical practice where ADHD may be the underlying cause of depressive symptoms yet remains unrecognized. This aligns with a previous Danish study [29], that showed common anti-depressive treatment prior to ADHD diagnosis. On the contrary, it is also possible that present findings (or at least part of it) could be explained by MDD symptoms mimicking ADHD symptoms. Mohr-Jensen et al. (2020) also reported a lifetime increase in treatment with anti-depressive drugs in ADHD patients [29].

Our study revealed several unexpected findings regarding symptom presentations in this undiagnosed population. Contrary to previous research, we found more males reporting inattentive symptoms and females more frequently reporting hyperactive-impulsive symptoms. This novel finding challenges existing literature and may reflect how ADHD presents differently in individuals who have developed successful coping strategies throughout life without a formal diagnosis. However, our finding of similar pADHD prevalence between the sexes supports previous findings suggesting that males exhibit higher prevalence in childhood, but that this may be due to underdiagnosis in females, which then narrows the prevalence gap in adulthood [30].

Furthermore, present findings suggest that the type of ADHD symptom presentation matters in terms of the level of MDD and MCS impact. We found that the non-specific presentation was the most common and that individuals classified with the combined inattentive and hyperactive-impulsive presentation experienced the highest burden associated with their experienced ADHD symptoms in terms of the highest risk of increased MDI score. Additionally, we observed the highest hazard ratio for later being prescribed anti-depressive medications for those with the inattentive presentation among males, whereas this was the case for the combined presentation among females. This finding may indicate that the impact on depressive symptoms varies depending on the expression of the experienced ADHD symptomatology. In any case, early diagnosis and initiation of ADHD treatment may reduce future comorbid depressive disorder [31], highlighting the importance of healthcare professionals not misattributing ADHD as depression. Moreover, it is relevant to point out that we identified physical health impacts in this otherwise healthy population. Individuals with pADHD were twice as likely to report low physical health compared to those below the ASRS cut-off, regardless of sex. This finding is particularly significant given our study population’s general good health status and suggests that even well-compensated ADHD symptoms may have broader health implications than previously recognized. It is also possible that such impairment could overshadow the ADHD symptoms, thus making it more difficult to obtain appropriate (ADHD) diagnosis and treatment [10, 32].

### Strengths and limitations

The blood donor population represents both a strength and limitation of this study. While the Healthy Donor Effect may limit generalizability [14, 33], this population provides unique insights into ADHD manifestation in high-functioning adults who developed effective coping strategies. The cohort’s health requirements and regular commitments make our findings particularly relevant for understanding ADHD in well-functioning populations. While our study identifies a subset of participants with elevated ADHD symptoms using the ASRS screening tool, we emphasize that this does not constitute a clinical diagnosis. The ASRS is a validated screening instrument with high sensitivity (0.92) and specificity (0.69), but it cannot replace comprehensive clinical assessment. Our findings should be interpreted as exploring the potential impact of elevated ADHD symptoms in a high-functioning population, rather than definitively identifying undiagnosed ADHD cases. In line with this, it is important to note that even though this study excluded individuals with a history of medically treated ADHD, the exclusion criteria focused on medical ADHD treatment but did not comprehensively assess prior clinical diagnoses and addressed the possibility of individuals with a non-medically treated ADHD diagnosis among participants.

Further, the cross-sectional design prevents causal inference, though our longitudinal analysis of anti-depressive medication provides temporal insights. This mixed approach helps balance the limitations of cross-sectional data while offering some perspective on temporal relationships between ADHD symptoms and depression. Self-reported data introduces potential misclassification bias, though validated questionnaires and objective prescription data mitigate this concern. Previous research suggests blood donors have no incentive to falsify information, though adults with ADHD may underreport symptom severity. Our use of multiple data sources helps address these potential limitations. Being unable to account for other psychiatric diagnoses limits our ability to determine whether impairments result from ADHD alone or condition combinations. However, individuals with a high psychiatric diagnosis burden likely wouldn’t become blood donors initially, providing some natural control for severe psychiatric comorbidity.

Our findings suggest significant ADHD underdiagnosis among Danish adults, even in well-functioning populations. The present study challenges traditional interpretations of the ADHD diagnosis impairment criterion. While participants meet strict blood donation health criteria, our findings reveal significant functional impairments across mental health and quality of life domains. The elevated ADHD symptom scores associated with lower mental component scores and increased depressive symptoms suggest that impairment can be subtle yet substantive, even in seemingly well-functioning adults. This underscores the complexity of ADHD manifestation, where individuals may develop sophisticated compensatory strategies that mask overt functional deficits while still experiencing meaningful internal challenges. Our results highlight that functional impairment should not be narrowly defined by external markers of success, but rather by comprehensive assessments of mental health, personal experience, and quality of life. To sum up, this study highlights the need for increased awareness of late-diagnosed ADHD and suggests current diagnostic practices may need revision to better identify affected individuals who developed effective compensatory strategies. The substantial impacts on mental and physical health, even in this healthy population, emphasize the importance of identifying and addressing ADHD symptoms regardless of apparent life function level.

These results may have important implications for clinical practice, suggesting the need for ADHD screening in patients presenting with depression, particularly in well-functioning adults who may have developed coping strategies that mask traditional ADHD presentations. Future research should examine the effectiveness of targeted screening approaches based on sex-specific presentation patterns and explore the mechanisms linking ADHD to physical health outcomes in otherwise healthy populations.

## Data Availability

Data can be made available upon reasonable request to info@dbds.dk (for more detail please see: https://bloddonor.dk/bloddonorstudiet/the-danish-blood-donor-study-eng/)
